# Strategies of neoadjuvant therapy in esophageal cancer: a study on the effects of treatment frequency and surgery interval

**DOI:** 10.3389/fonc.2025.1642765

**Published:** 2025-10-21

**Authors:** Huilai Lv, Bisong Chen, Chunyue Gai, Yu Liu, Weilu Ding, Mingbo Wang, Kangbo Sun, Fan Zhang, Yonggang Zhu, Zhenhua Li, Ziqiang Tian

**Affiliations:** ^1^ The Fourth Hospital of Hebei Medical University, Shijiazhuang, China; ^2^ Hebei Key Laboratory of Accurate Diagnosis and Comprehensive Treatment of Esophageal Cancer, Shijiazhuang, China

**Keywords:** esophageal squamous cell carcinoma, neoadjuvant chemoimmunotherapy, surgical interval, treatment cycles, disease-free survival

## Abstract

**Background:**

Neoadjuvant immunochemotherapy has shown remarkable efficacy in locally advanced ESCC. Therapy cycles and interval to surgery impact treatment efficacy. However, no consensus exists on the optimal cycles or surgical timing. This study investigates these factors to optimize perioperative management and improve patient outcomes.

**Methods:**

The postoperative follow-up data of 255 patients with esophageal cancer who were treated in the Department of Thoracic Surgery at the Fourth Hospital of Hebei Medical University from between November 6, 2019, and June 4, 2024, were retrospectively analyzed. A multivariate logistic regression with restricted cubic splines (RCS) was employed to model the association between the interval from treatment to surgery and primary outcomes.

**Results:**

All patients underwent neoadjuvant chemoimmunotherapy and 105 (41.1%) completed 2 cycles, 113 (44.3%) completed 3 cycles, and 37 (14.5%) completed 4 or more cycles. Most patients had an interval of 5 to 7 weeks between neoadjuvant therapy and surgery, with the highest proportion undergoing surgery at 6 weeks. Two cohorts were stratified by an interval of <6 weeks or ≥ 6 weeks with RCS. The short-interval group exhibited a higher survival probability in OS (P=0.059) and significantly better outcome in terms of DFS (P=0.018). The effect of different treatment cycles on survival outcomes revealed no significant differences in DFS (p=0.19) and OS (P=0.3).

**Conclusion:**

Surgical interval of less than six weeks following neoadjuvant chemoimmunotherapy is associated with improved disease-free survival and a trend toward better OS. While treatment cycle number exhibited no significant impact on survival. But these findings warrant further validation through multicenter prospective trials.

## 1 Introduction

Esophageal cancer is the 11th most common malignancy globally by incidence and the seventh leading cause of cancer-related deaths, with the highest incidence reported in China. Esophageal squamous cell carcinoma (ESCC) is the predominant subtype of esophageal cancer and is strongly associated with tobacco, alcohol consumption, and specific lifestyle factors. Notably, esophageal cancer demonstrates a higher prevalence in rural regions, which is largely attributed to dietary habits, water quality, and disparities in healthcare access (1). According to epidemiological data from 2022, esophageal cancer ranks fifth among cancer-related deaths in China and seventh in newly diagnosed cases. It is estimated to have caused 187,500 deaths and 224,000 new cases, accounting for 7.3% of total cancer-related deaths and 4.6% of total new cancer cases. High-incidence areas are predominantly concentrated in the provinces of Hebei, Henan, Shanxi, and Anhui (2).

Immune checkpoint inhibitors (ICIs), including PD-1/PD-L1 and CTLA-4 inhibitors, have significantly transformed cancer treatment paradigms over the past decade, achieving revolutionary advances particularly in the management of advanced and metastatic malignancies. Given their substantial therapeutic benefits in advanced-stage disease, ICIs are increasingly being explored investigated in perioperative settings, demonstrating considerable efficacy in neoadjuvant therapy, as supported by the ESCORT, ATTRACTION-3, KEYNOTE-181, KEYNOTE-590, and CheckMate-648 trials (3, 4). Moreover, neoadjuvant immunochemotherapy (NICT), which combines ICIs with neoadjuvant chemotherapy, has shown remarkable efficacy in locally advanced ESCC. This combinatory approach has contributed to a paradigm shift in the treatment strategies for multiple solid tumors, including non-small cell lung cancer and gastric cancer (5).

Multiple studies have shown that the interval between neoadjuvant chemoradiotherapy (nCRT) and surgery exerts a complex influence on efficacy in patients with esophageal cancer and gastroesophageal junction cancer. Klevebr’s research reported that extending the interval beyond 49 days neither improved the histological complete response rate (ypT0) nor enhanced postoperative survival outcomes (6). Azab’s study indicated that extending the interval could enhance the pathological complete response (pCR) rate, though exceeding 65 days was associated with higher 90-day mortality and reduced overall survival (OS) (7). Similarly, Lee et al. found that extending the interval from ≤40 days to ≥64 days increased the pCR rate from 12.3% to 18.3%, while it did not improve OS and was associated with a potential decline in OS and a rise in 90-day mortality (8). Haisley’s study indicated that an interval of 85 to 98 days was significantly associated with an increased likelihood of pCR, without any observed adverse effects on survival (9). Qin’s meta-analysis revealed that extending the time from nCRT to esophagectomy significantly improved the pCR rate in esophageal cancer, though it could negatively affect long-term survival (10).

Furthermore, similar interval timing effects have been studied in other types of cancer, such as rectal cancer, providing valuable insights into the optimal interval for neoadjuvant therapy across different cancer types. Sun’s study identified an 8-week interval as the critical threshold for achieving optimal tumor response and resection completeness (11). The study by Lefèvre J H demonstrated that extending the waiting period to four weeks post-chemoradiotherapy had no significant impact on oncological outcomes in T3/T4 rectal cancer patients (12). According to Probst C P’s study, extending the interval between nCRT and surgery beyond eight weeks in locally advanced rectal cancer significantly improved the pCR rate and tumor downstaging, without increasing postoperative complication risks. The optimal timing for surgery was estimated to be 10 to 11 weeks (13).

Regarding treatment cycles, no standardized regimen currently exists for neoadjuvant chemotherapy combined with immunotherapy in esophageal cancer, as previous studies have employed varying cycles, typically ranging from two to four (14–19). This variation may be influenced by multiple factors, including study design, differences in treatment protocols, patient tolerance, tumor biological characteristics, and considerations related to surgical timing. At present, the optimal cycle number for neoadjuvant chemotherapy combined with immunotherapy remains undetermined, and further research is required to evaluate the efficacy and safety of different regimens, with the goal of optimizing perioperative management and improving patient outcomes.

Esophageal cancer remains among the most lethal malignancies worldwide, with treatment outcomes and patient prognosis largely dependent on perioperative management. In recent years, neoadjuvant immunotherapy combined with chemotherapy has emerged as a promising treatment strategy for locally advanced esophageal cancer, demonstrating significant clinical potential. However, a consensus on the optimal surgical timing and the appropriate number of neoadjuvant immunotherapy cycles has yet to be established in clinical guidelines. The objective of this study is to comprehensively assess the effects of various optimal surgical timings and cycle numbers of neoadjuvant immunotherapy on prognosis, perioperative complications, and surgical outcomes in esophageal cancer patients. By identifying the ideal surgical timing and cycle number, we aim to refine perioperative management strategies, improve pCR rates and survival outcomes, and minimize postoperative complications.

## 2 Methods

### 2.1 Patients selection

This study was designed as a retrospective cohort study, enrolling adult patients with esophageal cancer who were treated in the Department of Thoracic Surgery at the Fourth Hospital of Hebei Medical University and underwent surgical resection following the completion of neoadjuvant immunotherapy between November 6, 2019, and June 4, 2024. The inclusion criteria were as follows: patients aged ≥18 years, histologically diagnosed with esophageal cancer, who received at least two cycles of neoadjuvant immunotherapy combined with chemotherapy (platinum- and taxane-based agents), and subsequently underwent surgical resection after completing neoadjuvant immunotherapy. The exclusion criteria included: patients diagnosed with two or more primary malignancies, those whose interval between the final administration of neoadjuvant therapy for esophageal cancer and surgery exceeded 16 weeks (potentially due to surgical delays for specific reasons), and patients lacking comprehensive clinical data or follow-up records. All enrolled patients underwent a standardized neoadjuvant immunotherapy-chemotherapy regimen, incorporating ICIs (such as PD-1 antibodies) in combination with chemotherapy agents, primarily platinum-based drugs (such as cisplatin or carboplatin) and taxane-based drugs (such as paclitaxel or docetaxel). Treatment was typically administered in three-week cycles, with a total of two to four cycles, adjusted according to the patient’s clinical condition and treatment tolerance.

### 2.2 Staging

To ensure precise clinical staging, comprehensive imaging assessments were conducted at baseline, prior to each cycle of neoadjuvant therapy, and preoperatively. These assessments included contrast-enhanced computed tomography (CT) scans of the chest and abdomen, contrast-enhanced esophageal magnetic resonance imaging (MRI), endoscopic ultrasound (EUS), and cervical ultrasound. Positron emission tomography (PET) was also utilized when clinically indicated. Postoperative pathological staging was conducted according to standard protocols, with both clinical and pathological staging determined based on the 8th edition of the American Joint Committee on Cancer (AJCC) TNM classification system.

### 2.3 Treatment and follow-up

Patients meeting the inclusion criteria underwent two to four cycles of neoadjuvant therapy, comprising a PD-1 inhibitor (200 mg, intravenous infusion, every three weeks [Q3W]) in combination with platinum- and taxane-based agents (intravenous infusion, Q3W). All candidates for curative-intent surgery underwent McKeown esophagectomy. Following surgery, patients were followed up every three months for the first two years and every six months thereafter.

### 2.4 Observation indices

pCR was defined as the absence of invasive cancer and high-grade intraepithelial neoplasia/severe dysplasia in both the primary tumor site and all sampled lymph nodes following neoadjuvant therapy, as determined by hematoxylin and eosin (H&E) staining, and classified as ypT0N0 based on the latest UICC/AJCC staging criteria. Major pathological response (MPR) was characterized by a residual tumor burden of ≤10% on pathological examination after neoadjuvant therapy-induced tumor regression. R0 resection referred to a microscopically margin-negative resection, indicating the absence of visible or microscopic tumor remnants within the primary tumor bed. Disease-free survival (DFS) was defined as the period from the first postoperative day to either disease recurrence or death from non-cancer-related causes. For patients without recurrence or metastasis, the last follow-up date or the time of non-cancer-specific death serves as the endpoint. OS was defined as the duration from the date of surgery to death due to any cause. The surgical interval was defined as the period between the last neoadjuvant therapy and surgery.

### 2.5 Statistical analysis

Multivariate logistic regression with restricted cubic splines (RCS) was employed to model the association between the interval from treatment to surgery and primary outcomes. RCS is a statistical method that partitions continuous variables along the x-axis and fits separate cubic polynomials to each segment, which are then smoothly connected to generate a regression curve that more accurately represents the data distribution. RCS allows for a flexible assessment of the relationship between continuous predictor variables and outcomes without imposing a predefined functional form. Prior research has demonstrated the utility of RCS in optimizing the timing of surgery following neoadjuvant chemoradiotherapy for esophageal adenocarcinoma and in identifying ideal candidates for capecitabine maintenance therapy in early-stage triple-negative breast cancer (20, 21). In this study, RCS was applied to model the association between surgical timing and primary as well as secondary outcomes, with graphical visualization of the RCS model to identify inflection points linked to pathological downstaging. After determining the inflection point, the cohort was stratified into two groups: a short-interval group (patients who underwent surgery before the inflection point) and a long-interval group (patients who underwent surgery after the inflection point) to evaluate the impact of interval duration on prognosis.

Continuous data were presented as mean ± standard deviation (SD) or median (interquartile range, IQR), with group comparisons performed using an independent samples t-test or Mann-Whitney U test. Categorical data were expressed as counts (percentages), with group differences assessed via the χ² test or Fisher’s exact test. Kaplan-Meier analysis was employed to evaluate DFS and OS, with survival curves presented along with 95% confidence intervals (CI), and group comparisons performed using the log-rank test. Subgroup analyses were performed based on key stratification variables (such as age, sex, and tumor stage) to determine whether treatment effects were consistent across patient subgroups. Cox proportional hazard regression was employed to compute hazard ratio (HR) with 95% CI for each subgroup, and interaction tests were used to evaluate statistical differences between them. Furthermore, multivariable Cox regression was performed to adjust for potential confounders, ensuring the independent evaluation of effects within subgroups. All statistical tests were two-sided, with P < 0.05 considered statistically significant. Statistical analyses were conducted using R software (version 4.4.2; R Foundation for Statistical Computing, Vienna, Austria).

## 3 Results

### 3.1 Baseline characteristics

This study enrolled a total of 255 esophageal cancer patients (**Table 1**), including 188 males (73.7%) and 67 females (26.3%), with a mean age of 64.3 years (SD: 6.91 years). Among these patients, 142 (55.7%) were non-smokers, and 134 (52.5%) were non-drinkers. The average height was 168cm (IQR:162–172 cm) and the mean weight of 65.4kg (SD: 10.7kg). In terms of clinical staging, the majority of patients were classified as cT3 (159 cases, 62.4%), cN1 (114 cases, 44.7%), and cM0 (248 cases, 97.3%). The predominant pathological type was squamous cell carcinoma (245 cases, 96.1%), followed by adenocarcinoma (8 cases, 3.14%) and small cell carcinoma (2 cases, 0.78%). The predominant clinical stage was stage III (113 cases, 44.3%). Tumors were primarily located in the middle thoracic esophagus (134 cases, 52.5%) and lower thoracic esophagus (96 cases, 37.6%). The median tumor length was 5.00cm (IQR: 4.00–6.00 cm). Preoperative response assessment revealed that the majority of patients exhibited a partial response (PR, 163 cases, 63.9%). The primary surgical approach was McKeown esophagectomy (231 cases, 90.6%). All patients underwent neoadjuvant chemoimmunotherapy. Of these, 105 (41.1%) completed 2 cycles, 113 (44.3%) completed 3 cycles, and 37 (14.5%) completed 4 or more cycles.

**Table 1:** Baseline Characteristics.

Characteristics	Patients (N=255), N (%)/Mean ± SD/Median (IQR)
Sex	
Male	188 (73.7%)
Female	67 (26.3%)
Age (year)	64.3 ± 6.91
Smoking history	
Non-smoker	142 (55.7%)
Smoker	113 (44.3%)
Alcohol consumption history	
Non-drinker	134 (52.5%)
Drinker	121 (47.5%)
Height (cm)	168 (162-172)
Weight (kg)	65.4 ± 10.7
cT	
T1	9 (3.53%)
T2	53 (20.8%)
T3	159 (62.4%)
T4a	27 (10.6%)
T4b	5 (1.96%)
Tis	2 (0.78%)
cN	
N0	86 (33.7%)
N1	114 (44.7%)
N2	49 (19.2%)
N3	2 (0.78%)
Nx	4 (1.57%)
cM	
M0	248 (97.3%)
M1	6 (2.35%)
Unknown	1 (0.39%)
Histological type	
Squamous cell carcinoma	245 (96.1%)
Adenocarcinoma	8 (3.14%)
Small cell carcinoma	2 (0.78%)
cTNM	
I	9 (3.53%)
II	91 (35.7%)
IIB	2 (0.78%)
III	113 (44.3%)
IVA	28 (11.0%)
IVB	6 (2.35%)
Unknown	6 (2.35%)
Tumor location by endoscopy	
Unknown	1 (0.39%)
Upper thoracic segment	24 (9.41%)
Lower thoracic segment	96 (37.6%)
Middle thoracic segment	134 (52.5%)
Tumor length by endoscopy (cm)	5.00 (4.00-6.00)
Number of treatment cycles	
>=4	37 (14.5%)
2	105 (41.2%)
3	113 (44.3%)
T stage & response (CE-CT)	
CR	26 (10.2%)
NA	19 (7.45%)
PD	3 (1.18%)
PR	163 (63.9%)
SD	44 (17.3%)
N stage & response (CE-CT)	
CR	17 (6.67%)
NA	25 (9.80%)
PD	2 (0.78%)
PR	121 (47.5%)
SD	90 (35.3%)

### 3.2 Restricted cubic splines

**Figure 1:**
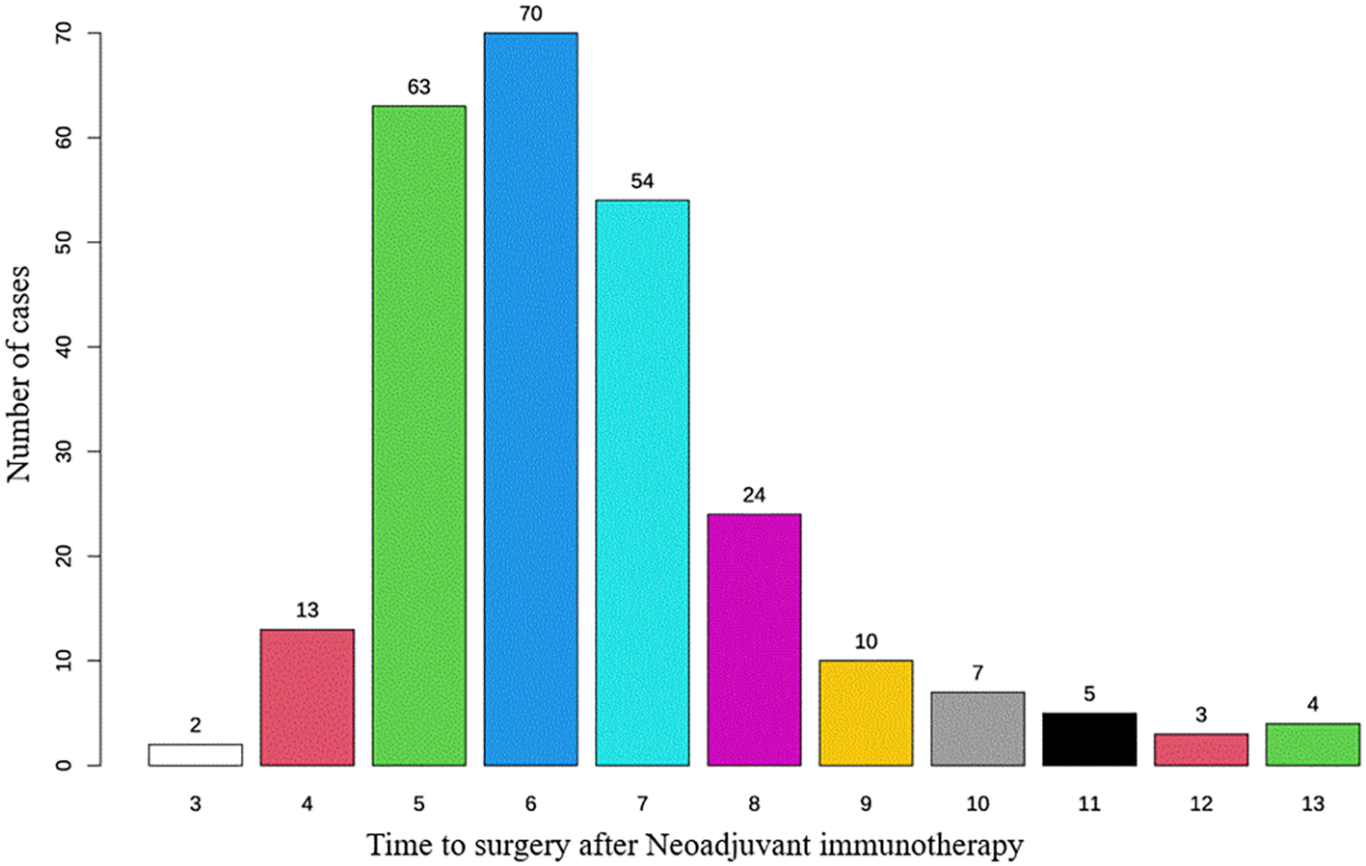
Distribution of time intervals for patients undergoing esophagectomy after neoadjuvant immunotherapy.

**Figure 2:**
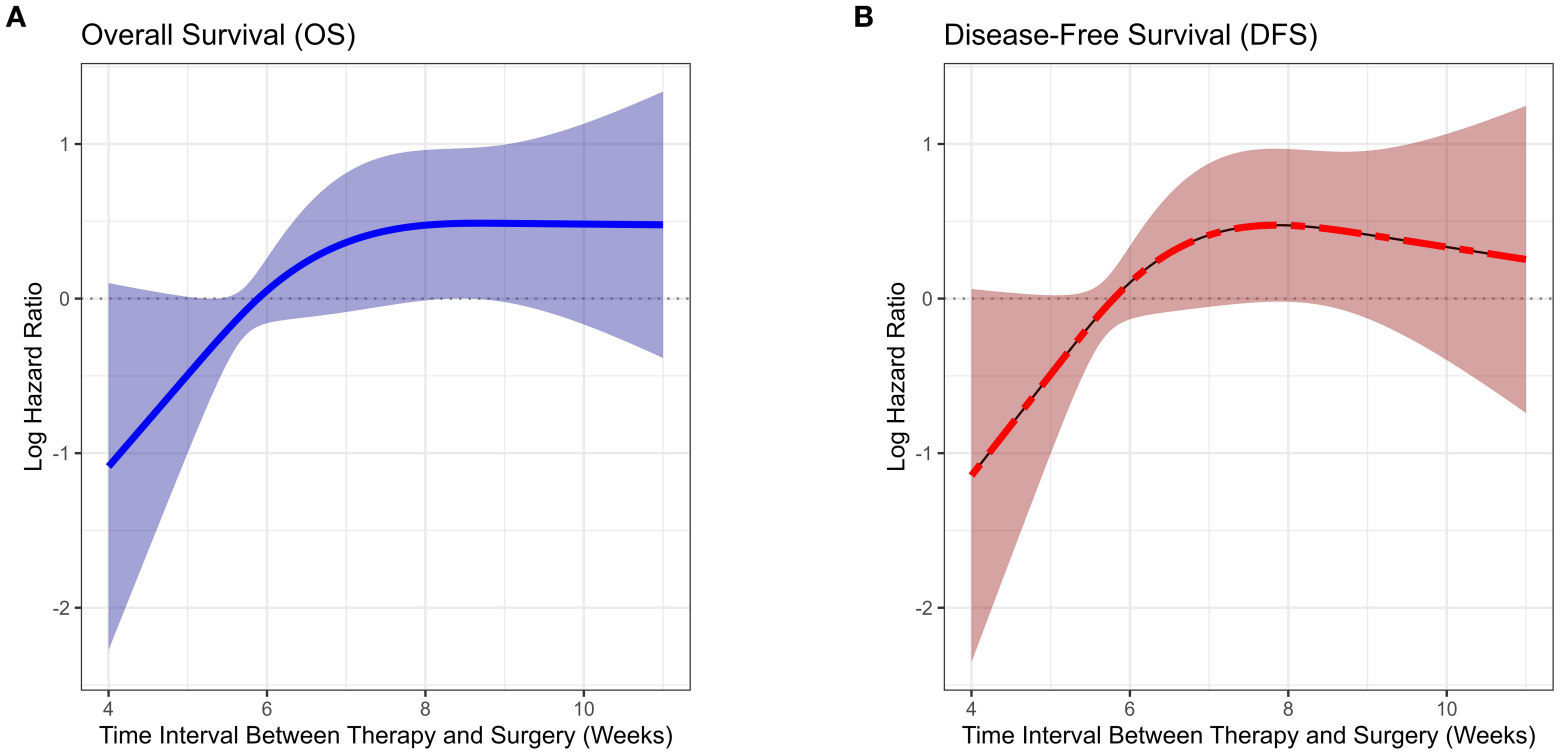
Association between time interval from neoadjuvant immunotherapy to surgery and survival risk (restricted cubic spline analysis).

Most patients had an interval of 5 to 7 weeks between neoadjuvant therapy and surgery, with the highest proportion undergoing surgery at 6 weeks, followed by 5 and 7 weeks, indicating a concentrated surgical scheduling pattern. With increasing interval duration, the number of patients declined, particularly among those undergoing surgery beyond 8 weeks. This finding indicates that, in routine clinical practice, most patients undergo surgery around 6 weeks after neoadjuvant therapy, whereas extended intervals (≥8 weeks) are relatively uncommon (**Figure 1**). Based on the RCS analysis, the survival risk (log HR) remained close to 0 and stabilized when the interval between neoadjuvant therapy and surgery ranged from 6 to 7 weeks (**Figure 2**). This suggests that within this time window, prolonging the interval had a diminishing impact on survival risk and was no longer statistically significant. This phenomenon was reflected in the survival curves of OS and DFS, showing a distinct demarcation. Given these findings, the study cohort was stratified into two groups: patients with an interval of less than 6 weeks and those with an interval of 6 weeks or more, allowing for a detailed evaluation of the impact of interval duration on prognosis.

### 3.3 Analysis of surgical outcomes and complications

The results of surgical outcomes and postoperative complications are displayed in **Table 2**. The median operative time was 313 minutes, and the median blood loss was 100 mL. Postoperative pathological examination confirmed that R0 resection was achieved in all cases. A pCR was observed in 30.2% (n=77) of patients (The absolute pCR rates for each cycle group and multivariable logistic regression for pCR, please see **Supplementary Tables 1**, **2**). The highest incidences of postoperative complications, including hoarseness and ineffective sputum clearance, were both 26.7% (68 cases). The incidences of other complications, including anastomotic leakage, tracheoesophageal fistula, chylothorax, interstitial pulmonary fibrosis, respiratory failure, mechanical ventilation, arrhythmia and anastomotic stricture, were 1.57% (4 cases), 0.78% (2 cases), 1.57% (4 cases), 2.35% (6 cases), 9.80% (25 cases), 20.0% (51 cases), 6.27% (16 cases), and 1.96% (5 cases), respectively. The mortality rate associated with complications was 0.39% (1 case).

**Table 2:** Surgical outcomes and complications.

Variables	N (%)/Median (IQR)
Surgical approach	
Ivor-Lewis	23 (9.02%)
McKeown	231 (90.6%)
Pneumomediastinoscopic	1 (0.39%)
Duration of surgery (min)	313 (284-355)
Intraoperative blood loss (mL)	100 (80.0-150)
Resection status: R0	255 (100%)
pcr(T0N0)	
No	178 (69.8%)
Yes	77 (30.2%)
Hoarseness	
Postoperative tracheostomy	1 (0.39%)
No	186 (72.9%)
Yes	68 (26.7%)
Ineffective expectoration	
No	187 (73.3%)
Yes	68 (26.7%)
Anastomotic leak	
No	251 (98.4%)
Yes	4 (1.57%)
Tracheoesophageal fistula	
No	253 (99.2%)
Yes	2 (0.78%)
Chylothorax	
No	251 (98.4%)
Yes	4 (1.57%)
Pneumonia	
No	100 (39.2%)
Yes	155 (60.8%)
Pulmonary interstitial fibrosis	
No	249 (97.6%)
Yes	6 (2.35%)
Respiratory failure	
No	230 (90.2%)
Yes	25 (9.80%)
Mechanical ventilation required	
No	204 (80.0%)
Yes	51 (20.0%)
Arrhythmia	
No	239 (93.7%)
Yes	16 (6.27%)
Complication-related death	
No	254 (99.6%)
Yes	1 (0.39%)
Anastomotic stricture	
No	250 (98.0%)
Yes	5 (1.96%)

### 3.4 Impact of on therapeutic efficacy and perioperative outcomes

Based on the RCS curve analysis, Patients were divided into a short-term group (surgical interval <6 weeks) and a long-term group (surgical interval ≥6 weeks) (**Table 3**). Preoperative contrast-enhanced CT evaluation of treatment response revealed that, in terms of T staging, 59.0% (46 cases) of patients in the short-term group exhibited PR, which was slightly higher compared to 66.1% (117 cases) in the long-term group, but the PR differences between the two groups were not statistically significant. The rates of complete response in the short-term group and long-term group were 8.97% (7 cases) and 10.7% (19 cases), respectively, with minimal difference between the two groups. The incidence of progressive disease was 0% in the short-term group, compared to 1.69% (3 cases) in the long-term group, indicating that the short-term group may be more effective in controlling disease progression. In the treatment response evaluation for N staging, 41.0% (32 cases) of the short-term group showed PR, compared to 50.3% (89 cases) in the long-term group. Although the long-term group showed slightly better performance, the difference was not statistically significant (P=0.503). Stable disease was observed in 21.8% (17 cases) of the short-term group and 15.3% (27 cases) of the long-term group. Although not statistically significant, the short-term group exhibited a slight advantage in maintaining disease stability. A shorter surgical interval may enhance immune response and limit tumor adaptation, thereby suppressing disease progression. Conversely, a prolonged interval could facilitate tumor cell adaptation and resistance, potentially elevating the risk of disease progression. Regarding surgery-related data, there was no significant difference in operative time and blood loss between the short-term and long-term groups.

**Table 3:** Impact of on therapeutic efficacy and perioperative outcomes.

Variables	Short-interval group (N=78), N (%)/Mean ± SD/Median (IQR)	Long-interval group (N=177), N (%)/Mean ± SD/Median (IQR)	p.overall
Sex			0.355
Male	61 (78.2%)	127 (71.8%)	
Female	17 (21.8%)	50 (28.2%)	
Age (year)	63.1 ± 7.16	64.8 ± 6.76	0.094
Smoking history			0.572
Non-smoker	46 (59.0%)	96 (54.2%)	
Smoker	32 (41.0%)	81 (45.8%)	
Alcohol consumption history			0.685
Non-drinker	39 (50.0%)	95 (53.7%)	
Drinker	39 (50.0%)	82 (46.3%)	
Height (cm)	167 ± 6.73	167 ± 8.02	0.840
Weight (kg)	65.4 ± 9.87	65.5 ± 11.0	0.937
cT			0.243
T1	1 (1.28%)	8 (4.52%)	
T2	12 (15.4%)	41 (23.2%)	
T3	51 (65.4%)	108 (61.0%)	
T4a	12 (15.4%)	15 (8.47%)	
T4b	2 (2.56%)	3 (1.69%)	
Tis	0 (0.00%)	2 (1.13%)	
cN			0.452
N0	30 (38.5%)	56 (31.6%)	
N1	31 (39.7%)	83 (46.9%)	
N2	16 (20.5%)	33 (18.6%)	
N3	1 (1.28%)	1 (0.56%)	
Nx	0 (0.00%)	4 (2.26%)	
cM			0.566
M0	75 (96.2%)	173 (97.7%)	
M1	3 (3.85%)	3 (1.69%)	
Unknown	0 (0.00%)	1 (0.56%)	
Histological type			0.404
Squamous cell carcinoma	76 (97.4%)	169 (95.5%)	
Adenocarcinoma	1 (1.28%)	7 (3.95%)	
Small cell carcinoma	1 (1.28%)	1 (0.56%)	
cTNM			0.434
I	1 (1.28%)	8 (4.52%)	
II	29 (37.2%)	62 (35.0%)	
IIB	0 (0.00%)	2 (1.13%)	
III	32 (41.0%)	81 (45.8%)	
IVA	12 (15.4%)	16 (9.04%)	
IVB	3 (3.85%)	3 (1.69%)	
Unknown	1 (1.28%)	5 (2.82%)	
Tumor location by endoscopy			0.833
Unknown	0 (0.00%)	1 (0.56%)	
Upper thoracic segment	7 (8.97%)	17 (9.60%)	
Lower thoracic segment	27 (34.6%)	69 (39.0%)	
Middle thoracic segment	44 (56.4%)	90 (50.8%)	
Tumor length by endoscopy (cm)	6.10 ± 2.78	5.16 ± 1.61	0.010
Number of treatment cycles			0.964
>=4	12 (15.4%)	25 (14.1%)	
2	32 (41.0%)	73 (41.2%)	
3	34 (43.6%)	79 (44.6%)	
T stage & response (CE-CT)			0.385
CR	7 (8.97%)	19 (10.7%)	
NA	8 (10.3%)	11 (6.21%)	
PD	0 (0.00%)	3 (1.69%)	
PR	46 (59.0%)	117 (66.1%)	
SD	17 (21.8%)	27 (15.3%)	
N stage & response (CE-CT)			0.503
CR	5 (6.41%)	12 (6.78%)	
NA	10 (12.8%)	15 (8.47%)	
PD	0 (0.00%)	2 (1.13%)	
PR	32 (41.0%)	89 (50.3%)	
SD	31 (39.7%)	59 (33.3%)	
Duration of surgery (min)	332 ± 70.1	321 ± 65.3	0.213
Intraoperative blood loss (mL)	127 ± 146	175 ± 248	0.053
Pathological tumor size (longest diameter)	3.14 ± 1.19	3.04 ± 1.35	0.535
Postoperative pathological type			0.022
Squamous cell carcinoma	47 (60.3%)	133 (75.1%)	
No residual tumor at tumor bed	31 (39.7%)	41 (23.2%)	
Adenocarcinoma	0 (0.00%)	1 (0.56%)	
Small cell carcinoma	0 (0.00%)	2 (1.13%)	
R0	78 (100%)	177 (100%)	.
pCR (T0N0)			0.383
No	51 (65.4%)	127 (71.8%)	
Yes	27 (34.6%)	50 (28.2%)	
ypT			0.359
T0	31 (39.7%)	54 (30.5%)	
T1	7 (8.97%)	23 (13.0%)	
T2	7 (8.97%)	32 (18.1%)	
T3	19 (24.4%)	39 (22.0%)	
T4a	12 (15.4%)	20 (11.3%)	
T4b	1 (1.28%)	4 (2.26%)	
Tis	1 (1.28%)	5 (2.82%)	
Tumor stage shift			0.999
Downstaging	47 (60.3%)	105 (59.3%)	
No downstaging	31 (39.7%)	72 (40.7%)	
ypN			0.009
N0	60 (76.9%)	98 (55.4%)	
N1	10 (12.8%)	47 (26.6%)	
N2	6 (7.69%)	27 (15.3%)	
N3	2 (2.56%)	5 (2.82%)	
Lymph node stage shift			0.045
Downstaging	37 (47.4%)	59 (33.3%)	
No downstaging	41 (52.6%)	118 (66.7%)	
ypTNM			0.300
0	27 (34.6%)	50 (28.2%)	
I	8 (10.3%)	15 (8.47%)	
II	22 (28.2%)	40 (22.6%)	
IIIA	2 (2.56%)	17 (9.60%)	
IIIB	13 (16.7%)	38 (21.5%)	
IVA	6 (7.69%)	17 (9.60%)	
Hoarseness			0.915
Postoperative tracheostomy	0 (0.00%)	1 (0.56%)	
No	58 (74.4%)	128 (72.3%)	
Yes	20 (25.6%)	48 (27.1%)	
Ineffective expectoration			0.830
No	56 (71.8%)	131 (74.0%)	
Yes	22 (28.2%)	46 (26.0%)	
Anastomotic leak			0.087
No	75 (96.2%)	176 (99.4%)	
Yes	3 (3.85%)	1 (0.56%)	
Tracheoesophageal fistula			0.093
No	76 (97.4%)	177 (100%)	
Yes	2 (2.56%)	0 (0.00%)	
Chylothorax			0.087
No	75 (96.2%)	176 (99.4%)	
Yes	3 (3.85%)	1 (0.56%)	
Pneumonia			0.054
No	38 (48.7%)	62 (35.0%)	
Yes	40 (51.3%)	115 (65.0%)	
Pulmonary interstitial fibrosis			1.000
No	76 (97.4%)	173 (97.7%)	
Yes	2 (2.56%)	4 (2.26%)	
Respiratory failure			1.000
No	70 (89.7%)	160 (90.4%)	
Yes	8 (10.3%)	17 (9.60%)	
Mechanical ventilation required			0.292
No	66 (84.6%)	138 (78.0%)	
Yes	12 (15.4%)	39 (22.0%)	
Arrhythmia			1.000
No	73 (93.6%)	166 (93.8%)	
Yes	5 (6.41%)	11 (6.21%)	
Complication-related death			0.306
No	77 (98.7%)	177 (100%)	
Yes	1 (1.28%)	0 (0.00%)	
Anastomotic stricture			0.169
No	75 (96.2%)	175 (98.9%)	
Yes	3 (3.85%)	2 (1.13%)	

The median operative time was 332 minutes in the short-term group and 321 minutes in the long-term group (P=0.213). The median blood loss was 127 mL in the short-term group and 175 mL in the long-term group (P=0.053), a difference approaching significance, suggesting that a longer surgical interval may be associated with increased operative time and higher blood loss. Postoperative pathological analysis showed that squamous cell carcinoma was the predominant pathological type in both groups, and all cases achieved R0 resection. Regarding pCR, 34.6% (27 cases) of patients in the short-term group achieved pCR, compared to 28.2% (50 cases) in the long-term group, with no statistically significant difference (P=0.383). However, in terms of lymph node response (ypN), 76.9% (60 cases) of the short-term group experienced downstaging, compared to 55.4% (98 cases) in the long-term group (P=0.009).

Regarding perioperative complications, the incidence of anastomotic leakage was 3.85% (3 cases) in the short-term group and 0.56% (1 case) in the long-term group, with a near-significant difference (P=0.087). While the short-term group exhibited a higher incidence of anastomotic leakage, this may be influenced by factors such as preoperative treatment response, tumor size, and postoperative recovery. No statistically significant differences were observed between the short-term and long-term groups in terms of other perioperative complications, including the need for mechanical ventilation and respiratory failure.

### 3.5 Overall survival and prognostic outcomes

By the data cutoff date, the median follow-up duration for the study cohort was 21.2 months (IQR: 8.9–28.9 months). During the follow-up period, overall patient survival was favorable. The 2-year DFS rate was 80.1% (95% CI, 74.4%–86.2%), indicating that most patients did not experience disease recurrence or progression postoperatively. Meanwhile, the 2-year OS rate was 82.8% (95% CI: 77.2%–88.3%), demonstrating the promising potential of neoadjuvant chemoimmunotherapy in improving short-term survival outcomes.

### 3.6 Impact of surgical interval on DFS and OS

**Figure 3:**
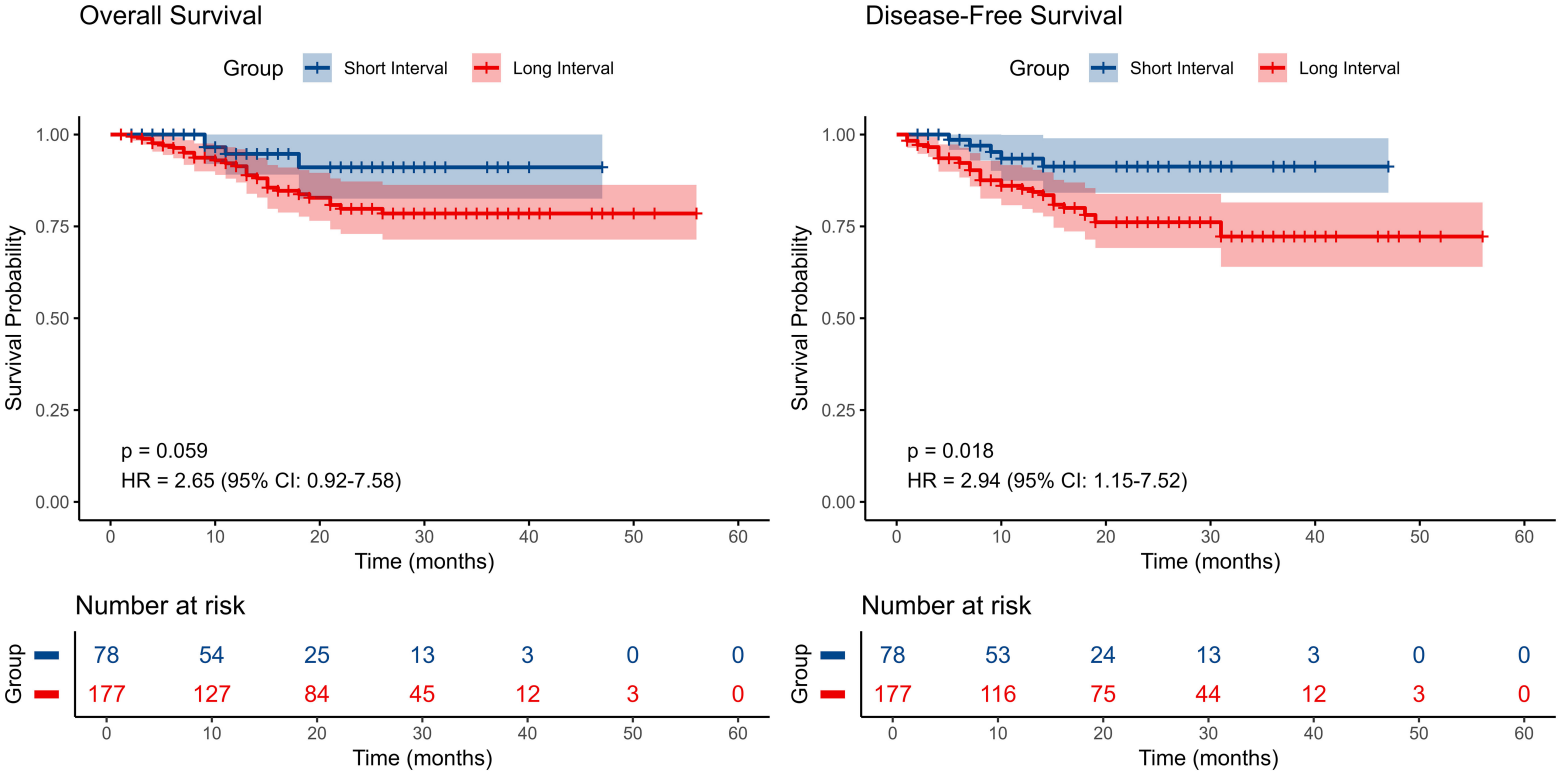
Kaplan–Meier survival curves comparing disease-free survival (DFS) and overall survival (OS) among patients with different surgical time intervals..

Kaplan-Meier survival analysis demonstrated that the short-interval group (neoadjuvant therapy to surgery <6 weeks) exhibited a higher survival probability in OS compared to the long-interval group (≥6 weeks), although the difference did not reach statistical significance (P=0.059) (**Figure 3**). However, in terms of DFS, the short-interval group achieved significantly better survival outcomes compared to the long-interval group (P=0.018). A more robust measure of long-term benefit by restricted mean survival time (RMST) is shown in **Supplementary Table 5**. These findings suggest that a shorter surgical interval may confer a prognostic advantage, particularly in reducing the risk of disease recurrence.

### 3.7 Impact of treatment cycles on DFS and OS

**Figure 4:**
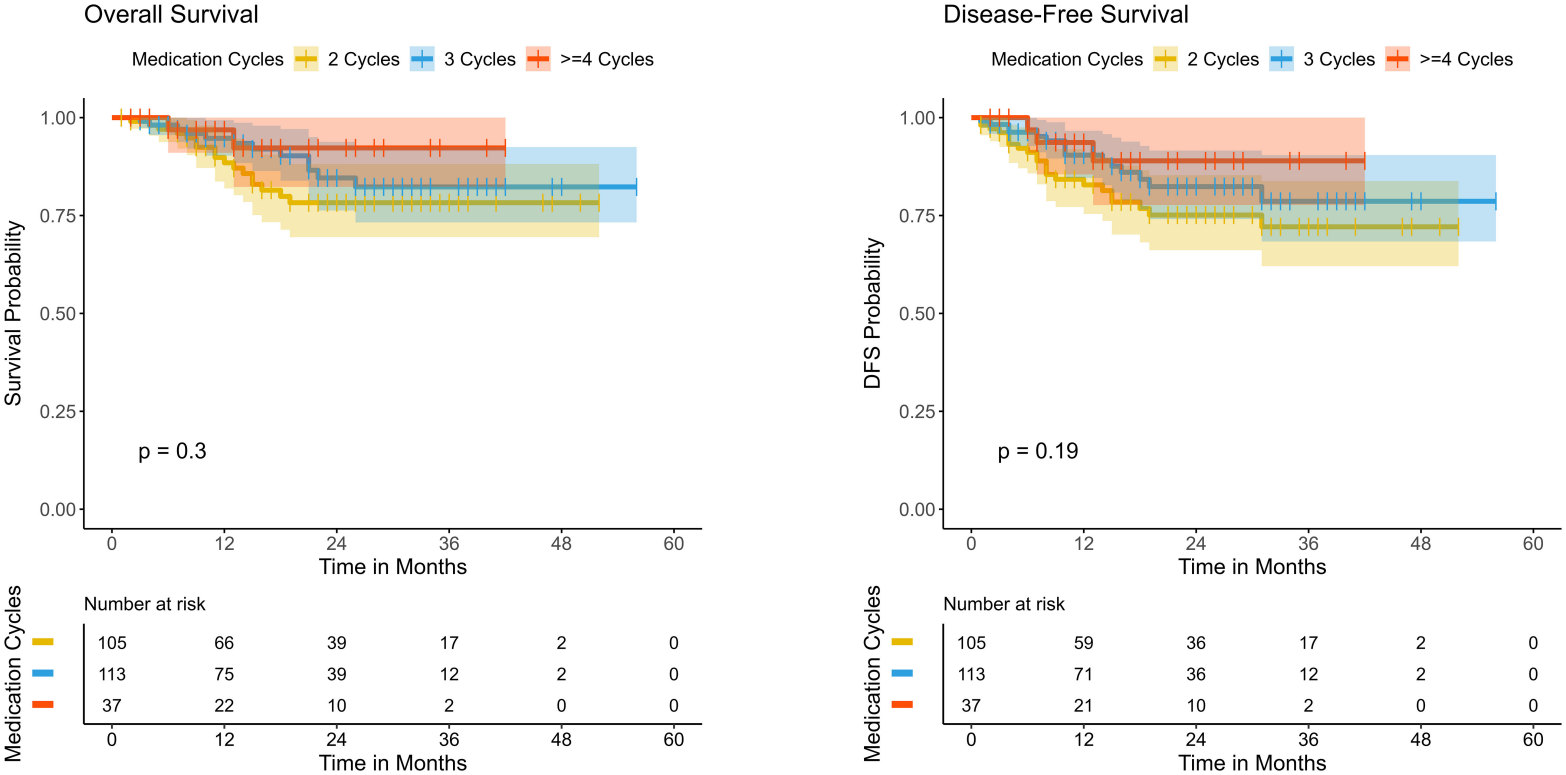
Kaplan–Meier survival curves comparing disease-free survival (DFS) and overall survival (OS) among patients receiving different cycles of neoadjuvant immunotherapy..

Further analysis of the effect of different treatment cycles on survival outcomes revealed no significant differences in DFS and OS among the three patient groups (**Figure 4**). Regarding DFS, the 4-cycle group (>=4 cycles) initially exhibited a relatively higher survival rate initially, followed by a slight decline, whereas the 2-cycle and 3-cycle groups showed a gradual decrease over time with a stable overall trend. The differences among groups were not statistically significant (P=0.19), indicating that the number of treatment cycles had a limited impact on DFS. For OS, the 4-cycle group initially demonstrated a higher survival rate, but this gradually declined over time. The 2-cycle and 3-cycle groups followed a similar trajectory. The differences among groups did not reach statistical significance (P=0.3), reinforcing the notion that treatment cycle count might have minimal influence on OS. Multivariable analysis by using a Cox proportional hazards model was also conducted to evaluate the association between the number of treatment cycles and DFS/OS, adjusting for age, sex, clinical T/N/Mstage, and surgery interval. The results indicated that the number of treatment cycles was not significantly associated with DFS or OS (**Supplementary Tables 3, 4**). These findings collectively suggest that the impact of treatment cycles on survival outcomes is limited.

### 3.8 Subgroup analysis

Subgroup analyses (**Tables 4** and **5**) indicated that a surgery interval of <6 weeks was associated with improved DFS (HR=2.96, 95% CI: 1.16-7.56, p=0.023). Overall survival (OS) showed a concordant trend but did not reach statistical significance (HR=2.65, 95% CI: 0.93-7.60, p=0.069). In stratified analyses, statistically significant DFS differences were observed in the following subgroups: male sex (HR=2.95, 95% CI: 1.03-8.47, p=0.044), cT3-4 (HR=2.99, 95% CI: 1.15-7.76, p=0.024), cN1-3 (HR=5.86, 95% CI: 1.40-24.55, p=0.016), clinical stage III–IV (HR=3.95, 95% CI: 1.19-13.13, p=0.025), M0 (HR=2.92, 95% CI: 1.14-7.47, p=0.025), squamous cell carcinoma (HR=3.06, 95% CI: 1.20-7.81, p=0.020), and postoperative ypT3-4 (HR=3.03, 95% CI: 1.04-8.79, p=0.042). Patients without a pathological complete response (pCR) likewise demonstrated a significant difference (HR=3.25, 95% CI: 1.15-9.18, p=0.026). The corresponding OS effects in these key subgroups were generally directionally consistent but mostly nonsignificant, suggesting that larger cohorts or longer follow-up are required for confirmation. Notably, the number of treatment cycles showed no significant interaction with either OS or DFS (interaction p=0.565 for OS; p=0.42 for DFS), indicating a limited impact of cycle number on survival outcomes.

**Table 4:** Subgroup analysis on overall survival.

Variable	Count (N)	Percentage (%)	HR	Lower	Upper	P value
Overall	251	100	2.65	0.93	7.6	0.069
Age						
<65	119	47.4	2.16	0.48	9.81	0.318
>=65	132	52.6	2.95	0.68	12.84	0.149
Gender						
Male	185	73.7	3.22	0.96	10.76	0.058
Female	66	26.3	1.27	0.15	10.93	0.83
Smoking history						
Non-smoker	140	55.8	2.04	0.45	9.22	0.356
Smoker	111	44.2	3.26	0.75	14.19	0.115
Alcohol consumption history						
Non-drinker	133	53	4.5	0.58	34.68	0.149
Drinker	118	47	2.13	0.62	7.39	0.232
BMI						
BMI<18.5	12	4.8	3.29	0.38	28.41	0.28
18.5 ≤ BMI <24.0	132	52.6	2.4	0.69	8.3	0.167
cT						
T3T4	188	74.9	2.77	0.95	8.05	0.061
cN						
N0	86	34.3	1.03	0.19	5.64	0.97
N1N2N3	165	65.7	4.18	0.98	17.72	0.053
cM						
M0	245	97.6	2.63	0.92	7.53	0.071
M1	6	2.4				
cTNM staging						
I-II	102	40.6	2.7	0.34	21.47	0.347
III-IV	147	58.6	2.76	0.81	9.38	0.104
Unknown	2	0.8				
Pathology						
Squamous cell carcinoma	241	96	2.74	0.96	7.83	0.06
Adenocarcinoma	8	3.2				
Small cell carcinoma	2	0.8				
Treatment cycles						
2	105	41.8	1.98	0.57	6.89	0.284
3	109	43.4	3.25	0.41	25.41	0.262
pCR						
N	174	69.3	3.24	0.98	10.7	0.054
Y	77	30.7	0.51	0.03	8.09	0.63
ypT						
T0T1T2	158	62.9	3.51	0.45	27.5	0.231
T3T4	93	37.1	2.71	0.79	9.26	0.112
Tumor stage progression						
Down-staging	96	38.2	2.89	0.34	3.1	0.334
No down-staging	155	61.8	2.09	0.62	16.2	0.233
ypN						
N0N1	213	84.9	2.89	0.86	9.73	0.087
N2N3	38	15.1	1.19	0.14	9.77	0.872
N progression						
Down-staging	96	38.2	2.89	0.34	24.73	0.334
No down-staging	155	61.8	2.09	0.62	7	0.233
ypTNM						
0-II	160	63.7	3.51	0.43	28.62	0.24
III-IV	91	36.3	1.64	0.49	5.53	0.427

**Table 5:** Subgroup analysis on disease-free survival.

Variable	Count (N)	Percentage (%)	HR	Lower	Upper	P value
Overall	251	100	2.96	1.16	7.56	0.023
Age						
<65	119	47.4	3.17	0.71	14.1	0.129
>=65	132	52.6	2.74	0.82	9.17	0.101
Gender						
Male	185	73.7	2.95	1.03	8.47	0.044
Female	66	26.3	3	0.38	23.8	0.297
Smoking history						
Non-smoker	140	55.8	2.24	0.65	7.67	0.199
Smoker	111	44.2	3.9	0.9	16.8	0.068
Alcohol consumption history						
Non-drinker	133	53	6.46	0.86	48.79	0.07
Drinker	118	47	2.06	0.7	6.09	0.192
BMI						
BMI < 18.5	12	4.8	3.02	0.35	26.13	0.316
18.5 ≤ BMI < 24.0	132	52.6	2.34	0.79	6.88	0.123
cT						
T3T4	188	74.9	2.99	1.15	7.76	0.024
cN						
N0	86	34.3	0.83	0.2	3.44	0.798
N1N2N3	165	65.7	5.86	1.4	24.55	0.016
cM						
M0	245	97.6	2.92	1.14	7.47	0.025
M1	6	2.4				
cTNM staging						
I-II	102	40.6	1.7	0.37	7.78	0.494
III-IV	147	58.6	3.95	1.19	13.13	0.025
Unknown	2	0.8				
Pathology						
Squamous cell carcinoma	241	96	3.06	1.2	7.81	0.02
Adenocarcinoma	8	3.2				
Small cell carcinoma	2	0.8				
Treatment cycles						
2	105	41.8	2.91	0.86	9.82	0.086
3	109	43.4	2.22	0.5	9.91	0.298
pCR						
N	174	69.3	3.25	1.15	9.18	0.026
Y	77	30.7	1.11	0.11	11.1	0.932
ypT						
T0T1T2	158	62.9	4.72	0.62	36.16	0.135
T3T4	93	37.1	3.03	1.04	8.79	0.042
Down-staging	68	27.1	2.93	0.34	2.8	0.327
No down-staging	183	72.9	2.35	0.83	15.3	0.109
ypN						
N0N1	213	84.9	2.5	0.86	7.26	0.092
N2N3	38	15.1	2.59	0.34	19.88	0.361
N progression						
Down-staging	96	38.2	2.93	0.34	25.12	0.327
No down-staging	155	61.8	2.35	0.83	6.7	0.109
ypTNM						
0-II	160	63.7	4.51	0.57	35.67	0.153
III-IV	91	36.3	1.82	0.63	5.24	0.264

## 4 Discussion

In recent years, neoadjuvant chemoimmunotherapy has demonstrated remarkable potential in the management of locally advanced esophageal cancer. However, the optimal of the interval between preoperative therapy and surgery and its effects on perioperative care and patient prognosis, both short- and long-term, remain a topic of debate. The NCCN guidelines recommend performing evaluation and surgery at least 5 to 8 weeks post-neoadjuvant chemoradiotherapy, whereas the 2024 Chinese Society of Clinical Oncology (CSCO) esophageal cancer guidelines recommend surgery at 4–8 weeks after chemoradiotherapy or 3–6 weeks following chemotherapy alone. Similarly, the Chinese perioperative treatment guidelines for resectable esophageal cancer state that surgery can be planned 3–6 weeks after neoadjuvant chemotherapy. However, ICIs function by regulating the immune system, a mechanism distinct from traditional therapies, thus posing new challenges in optimizing surgical timing. Utilizing real-world data, this study evaluates the effect of the interval between neoadjuvant therapy and surgery on perioperative management and patient prognosis, particularly in China, a high-incidence region for esophageal cancer. By systematically comparing short- and long-interval groups, this research seeks to establish scientific evidence for optimal surgical timing after neoadjuvant immunotherapy, optimize clinical decision-making, refine personalized treatment strategies, and enhance patient outcomes and quality of life.

This study demonstrated that after implementing a neoadjuvant treatment regimen, the 2-year OS rate was 82.8%, and the DFS rate was 80.1%, indicating significant survival benefits from this therapeutic approach. These findings are consistent with the 91% OS and 89% DFS rates reported in the Keystone-001 study (18), further supporting the validity and reproducibility of our results. Furthermore, this study provides evidence for the potential of neoadjuvant chemoimmunotherapy to improve survival outcomes in esophageal cancer patients. Notably, the pCR rate in this study reached 30.2%. Based on several studies, the pCR rate among patients with varying surgical intervals (2–14 weeks) has consistently ranged between 28.1% and 44% (22–27). All enrolled patients successfully completed surgery, achieving a 100% R0 resection rate, suggesting that the neoadjuvant treatment strategy played a crucial role in reducing tumor burden and improving the radicality of surgery.

To further investigate the impact of surgical interval on perioperative outcomes and postoperative survival prognosis, this study stratified patients into a short-term group (surgical interval <6 weeks) and a long-term group (surgical interval ≥6 weeks). There was a near-significant difference in intraoperative blood loss between the two groups (P=0.053), suggesting that prolonged surgical waiting time may increase surgical complexity, while a shorter interval might contribute to reducing intraoperative bleeding risk. Liang et al. (28) reported that immunotherapy could induce significant tissue edema, obscuring tissue planes and increasing surgical complexity. Additionally, prolonging the interval between neoadjuvant therapy and surgery may exacerbate tissue fibrosis, thereby adversely affecting the surgical process (29). Although the incidence of anastomotic leakage was slightly higher in the short-term group compared to the long-term group (3.85% vs. 0.56%, P=0.087), no significant differences were observed between the two groups in other perioperative complications such as the need for mechanical ventilation or respiratory failure (p>0.05), indicating that the surgical interval had a limited impact on overall perioperative safety.

Regarding survival analysis, the short-term group demonstrates a notable advantage in DFS (HR=2.94, 95% CI: 1.15-7.52, p=0.018), whereas for OS, the difference between the two groups approached statistically significant (HR=2.65, 95% CI: 0.92-7.58, P=0.07). Accelerated repopulation represents a significant factor in radiotherapy failure, especially in squamous cell carcinoma, as surviving tumor cells can rapidly proliferate during the intervals between treatments, thus impacting the effectiveness of the therapy (30). Surgical procedures should not be excessively delayed, as this could result in missing the optimal window for clearance, thereby causing residual cells to grow at an accelerated rate. Additional studies indicate that in the course of intermittent therapy, the surviving resistant cells can multiply during the gaps between treatments and can further increase their resistance via genetic mutations or epigenetic modifications. Hence, prolonged intervals between treatments could inadvertently facilitate the accumulation of resistant clones, rendering the relapsed cancer more difficult to manage (31). Additionally, extended delays before surgery can lead to muscle deterioration, which in turn impacts the patient’s recovery after surgery and the development of complications (32). Notably, neoadjuvant treatment boosts anti-tumor immunity within a short timeframe; however, it also suggests that excessively long treatment intervals could lead to the reactivation of immune escape mechanisms, allowing surviving cancer cells to restore their immune evasion through the regulation of immune checkpoints or other suppressive pathways, thus diminishing the immune system’s cytotoxic capacity (33).

Additional subgroup analysis revealed that patients in more advanced stages (cT3-T4, cN1-N3, cTNM III-IV) and those not achieving pCR experienced significantly prolonged DFS in the short surgery interval group (p<0.05), indicating a potentially greater survival advantage for these patients with this approach. Nevertheless, regarding OS, although patients not achieving pCR demonstrated a tendency toward longer survival times in the short surgery interval group, this difference was not statistically significant when compared to the long interval group (P=0.055). Moreover, the analysis of treatment cycles revealed no statistically significant differences in OS (P=0.59) or DFS (P=0.42) across different cycle numbers, suggesting that the duration of treatment cycles might have a minimal effect on survival outcomes.

This study has the following limitations. First, as a single-center retrospective study, our research is potentially subject to selection bias and immortal-time bias, which may limit the external validity of the results. For instance, referral patterns or treatment delays could have influenced the interval from the last NICT to surgery. Moreover, patients who died from rapid disease progression or toxicity during this waiting period would have been excluded from the study cohort since they did not undergo surgery, further contributing to immortal-time bias. Therefore, future multi-center prospective studies, particularly those using standardized protocols through prospective registries or multi-center collaborations, are essential to control these biases, enhance the generalizability and robustness of the results, and determine the true optimal timing for surgery after NICT. Second, the follow-up period of 21.2 months (IQR: 8.9–28.9 months) is relatively short for EC, a disease often associated with late recurrences, and the median follow-up time may be insufficient to fully evaluate long-term survival outcomes, particularly regarding distant recurrence and long-term prognosis in advanced-stage patients. Therefore, future analyses with extended prospective follow-up are essential to validate whether a shorter surgical interval confers sustained oncologic benefits. Third, the sample size of this study is relatively small and shows a substantial imbalance (177 in the long-interval group vs. 78 in the short-interval group), which may limit the statistical power. Notably, although the OS analysis suggested a trend toward a difference between the two surgery interval groups, the result did not reach statistical significance (P=0.059). A *post-hoc* power calculation for this comparison, assuming a HR of 2.64 and a significance level of α = 0.05, yielded a power of 50.3%, further underscoring the need for caution in interpreting the non-significant trend. Therefore, larger and more balanced cohort studies are warranted in the future to validate the robustness of these findings. Fourth, this study did not capture immune-related adverse events (irAEs) or their timing, preventing assessment of whether treatment delays due to toxicity influenced the interval-outcome relationship. However, the low rate of postoperative complications and very low mortality (0.39%) suggest that severe preoperative toxicity was uncommon. Therefore, while residual confounding from irAEs cannot be excluded, the findings likely reflect the effect of surgical timing itself rather than to a burden of pre-operative toxicity. In summary, although this study provides preliminary evidence for optimizing the surgery interval to improve survival outcomes in esophageal cancer patients, our analysis did not incorporate key molecular variables, such as tumor mutational burden, PD-L1 expression, or genetic alterations, which may influence both treatment sensitivity and the optimal timing of surgery. This limitation underscores the need to integrate biomarker data in future prospective studies to enable more personalized surgical timing strategies, thereby guiding individualized treatment. Long-term follow-up studies are also essential to clarify 3–5-year survival rates, patterns of distant metastasis, and postoperative recurrence risks, thereby optimizing treatment strategies and improving long-term patient benefits.

## 5 Conclusions

This study demonstrates that a surgical interval of less than six weeks following neoadjuvant chemoimmunotherapy is associated with improved disease-free survival and a trend toward better OS, particularly in advanced-stage esophageal cancer. While treatment cycle number exhibited no significant impact on survival, prolonged intervals did not confer additional benefits and may potentially increase surgical complexity. These findings underscore the need for optimized perioperative strategies to prevent tumor adaptation and immune evasion. Despite the study’s retrospective nature and limited follow-up duration, it provides compelling evidence for refining surgical timing, warranting further validation through multicenter prospective trials.

## Data Availability

The raw data supporting the conclusions of this article will be made available by the authors, without undue reservation.
