# Supplementary material for: Strategies of neoadjuvant therapy in esophageal cancer: a study on the effects of treatment frequency and surgery interval
Source: Front Oncol. 2025 Oct 21;15:1642765. doi: 10.3389/fonc.2025.1642765 (PMC12603392; doi:10.3389/fonc.2025.1642765)

**Supplementary Table 1**: pCR rates for each cycle group

| **Treatment cycles** | **pCR rates** | **non-pCR rates** | **Patient No. in each group** | **Test Statistic** | **P value** |
| --- | --- | --- | --- | --- | --- |
| 2 | 25 (23.81%) | 80(44.94%) | 105 | 3.570 | 0.1678 |
| 3 | 40 (35.40%) | 73(41.01%) | 113 |  |  |
| 4 | 12 (32.43%) | 25(14.04%) | 37 |  |  |

**Supplementary Table 2**: Multivariable logistic regression for pCR

| Term | OR | CI.Lower | CI.Upper | P value |
| --- | --- | --- | --- | --- |
| (Intercept) | 0.309 | 0.018 | 5.175 | 0.415 |
| Treatment cycles -3 | 0.537 | 0.287 | 0.988 | 0.048* |
| Treatment cycles -4 | 0.585 | 0.251 | 1.398 | 0.218 |
| Surgery long-interval | 1.321 | 0.715 | 2.421 | 0.370 |
| Age | 1.022 | 0.980 | 1.066 | 0.312 |
| Sex-female | 0.837 | 0.445 | 1.601 | 0.585 |
| T stage cT3-4 | 2.140 | 1.142 | 4.007 | 0.017* |
| N stage cN1-3 | 1.685 | 0.933 | 3.041 | 0.083 |
| M stage M1 | 0.170 | 0.022 | 0.971 | 0.055 |

**Supplementary Table 3**: Multivariable Cox regression for DFS

| Term | HR | 95%CI_low | 95%CI_high | P value |
| --- | --- | --- | --- | --- |
| Treatment cycles -3 | 0.599 | 0.308 | 1.161 | 0.129 |
| Treatment cycles -4 | 0.379 | 0.112 | 1.279 | 0.118 |
| Surgery long-interval | 3.271 | 1.265 | 8.460 | 0.015* |
| Age | 1.015 | 0.966 | 1.06 | 0.556 |
| Sex-female | 0.869 | 0.423 | 1.786 | 0.703 |
| T stage cT3-4 | 1.880 | 0.822 | 4.302 | 0.135 |
| N stage cN1-3 | 1.696 | 0.802 | 3.586 | 0.167 |
| M stage M1 | 0.000 | 0.000 | Inf | 0.997 |

**Supplementary Table 4**: Multivariable Cox regression for OS

| Term | HR | 95%CI_low | 95%CI_high | P value |
| --- | --- | --- | --- | --- |
| Treatment cycles -3 | 0.636 | 0.302 | 1.340 | 0.234 |
| Treatment cycles -4 | 0.358 | 0.082 | 1.563 | 0.172 |
| Surgery long-interval | 2.970 | 1.024 | 8.611 | 0.045* |
| Age | 1.017 | 0.962 | 1.074 | 0.557 |
| Sex-female | 0.598 | 0.244 | 1.464 | 0.260 |
| T stage cT3-4 | 2.006 | 0.761 | 5.287 | 0.159 |
| N stage cN1-3 | 1.929 | 0.786 | 4.734 | 0.151 |
| M stage M1 | 0.000 | 0.000 | Inf | 0.996 |

**Supplementary Table 5**: Comparison of RMST for DFS and OS by surgery interval or treatment cycles.

|  | **RMST** | **long-interval** | **short-interval** | **RMST (arm=1)-(arm=0)** | **p_value** |
| --- | --- | --- | --- | --- | --- |
| **Comparison of RMST for DFS by Surgery Interval** | 12-month RMST(95%CI) | 11.08  (10.71, 11.46) | 11.73 (11.46, 12.00) | -0.64(-1.11 ,-0.17) | 0.007 |
|  | 24-month RMST(95%CI) | 20.61 (19.55, 21.68) | 22.73 (21.65, 23.80) | -2.11 (-3.62, -0.60) | 0.006 |
|  | 36-month RMST(95%CI) | 29.55 (27.66, 31.45) | 33.68 (31.73, 35.63) | -4.12 (-6.84,-1.41) | 0.003 |
|  | **RMST** | **long-interval** | **short-interval** | **RMST (arm=1)-(arm=0)** | **p_value** |
| **Comparison of RMST for OS by Surgery Interval** | 12-month RMST(95%CI) | 11.57 (11.31, 11.82) | 11.88 (11.73, 12.02) | -0.31 (-0.60, -0.02) | 0.038 |
|  | 24-month RMST(95%CI) | 21.70 (20.84, 22.56) | 23.03 (22.11, 23.95) | -1.33 (-2.59, -0.07) | 0.038 |
|  | 36-month RMST(95%CI) | 31.14 (29.49, 32.80) | 33.96 (32.01, 35.91) | -2.81 (-5.37, -0.25) | 0.031 |
|  | **RMST** | **4** | **2** | **RMST (arm=1)-(arm=0)** | **p_value** |
| **Comparison of RMST for DFS by Treatment Cycles (2 and 4 groups)** | 12-month RMST(95%CI) | 11.65 (11.18, 12.12,) | 10.95(10.43 11.46) | 0.70(0.002 1.40) | 0.049 |
|  | 24-month RMST(95%CI) | 22.37 (20.63, 24.12) | 20.29 (18.86, 21.73) | 2.07 (-0.18, 4.33) | 0.072 |
|  | 36-month RMST(95%CI) | 33.04 (29.88, 36.22) | 29.16(26.64, 31.68) | 3.88 (-0.165, 7.983) | 0.06 |
|  | **RMST** | **2** | **3** | **RMST (arm=1)-(arm=0)** | **p_value** |
| **Comparison of RMST for DFS by Treatment Cycles (3 and 2 groups)** | 12-month RMST(95%CI) | 10.95  (10.43,11.46) | 11.48 (11.12 11.84) | -0.53 (-1.16, 0.09) | 0.096 |
|  | 24-month RMST(95%CI) | 20.29  (18.86, 21.73) | 21.74 (20.62, 22.86) | -1.44  (-3.26 0.37) | 0.120 |
|  | 36-month RMST(95%CI) | 29.16 (26.64, 31.68) | 31.44(29.32, 33.55) | -2.27 (-5.57, 1.01) | 0.175 |
|  | **RMST** | **4** | **3** | **RMST (arm=1)-(arm=0)** | **p_value** |
| **Comparison of RMST for DFS by Treatment Cycles (3 and 4 groups)** | 12-month RMST(95%CI) | 11.65 (11.18, 12.12) | 11.48 (11.12, 11.84) | 0.16 (-0.42, 0.75) | 0.583 |
|  | 24-month RMST(95%CI) | 22.37 (20.63, 24.12) | 21.74 (20.62, 22.86) | 0.63(-1.44, 2.70) | 0.551 |
|  | 36-month RMST(95%CI) | 33.05 (29.88, 36.22) | 31.41 (29.32, 33.55) | 1.60(-2.20, 5.42) | 0.409 |
|  | **RMST** | **4** | **2** | **RMST (arm=1)-(arm=0)** | **p_value** |
| **Comparison of RMST for OS by Treatment Cycles (2 and 4 groups)** | 12-month RMST(95%CI) | 11.81 (11.45, 12.17) | 11.56 (11.24, 11.88) | 0.26 (-0.23, 0.74) | 0.297 |
|  | 24-month RMST(95%CI) | 22.93 (21.50, 24.36) | 21.34 (20.17, 22.51) | 1.59 (-0.26, 3.44) | 0.092 |
|  | 36-month RMST(95%CI) | 34.00 (31.33, 36.68) | 30.73 (28.49, 32.98) | 3.27 (-0.22, 6.76) | 0.067 |
|  | **RMST** | **2** | **3** | **RMST (arm=1)-(arm=0)** | **p_value** |
| **Comparison of RMST for OS by Treatment Cycles (3 and 2 groups)** | 12-month RMST(95%CI) | 11.56 (11.24, 11.88) | 11.72 (11.46, 11.98) | -0.16 (-0.57, 0.25) | 0.441 |
|  | 24-month RMST(95%CI) | 21.34 (20.17, 22.51) | 22.56 (21.68, 23.44) | -1.22 (-2.68, 0.25) | 0.104 |
|  | 36-month RMST(95%CI) | 30.73 (28.49, 32.98) | 32.48 (30.63, 34.33) | -1.75 (-4.65, 1.16) | 0.239 |
|  | **RMST** | **4** | **3** | **RMST (arm=1)-(arm=0)** | **p_value** |
| **Comparison of RMST for OS by Treatment Cycles (3 and 4 groups)** | 12-month RMST(95%CI) | 11.81 (11.45, 12.17) | 11.72 (11.46, 11.98) | 0.10 (-0.35, 0.54) | 0.675 |
|  | 24-month RMST(95%CI) | 22.93 (21.50, 24.36) | 22.56 (21.68, 23.44) | 0.37 (-1.31, 2.05) | 0.664 |
|  | 36-month RMST(95%CI) | 34.00 (31.33, 36.68) | 32.48 (30.63, 34.33) | 1.52 (-1.73, 4.77) | 0.359 |

**Supplementary Figure 1**: Kaplan–Meier curves for DFS and OS by treatment cycle in the pCR and non-pCR populatioDn. A. DFS in the pCR population; B. OS in the pCR population; C. DFS in the non-pCR population; D. OS in the non-pCR population.
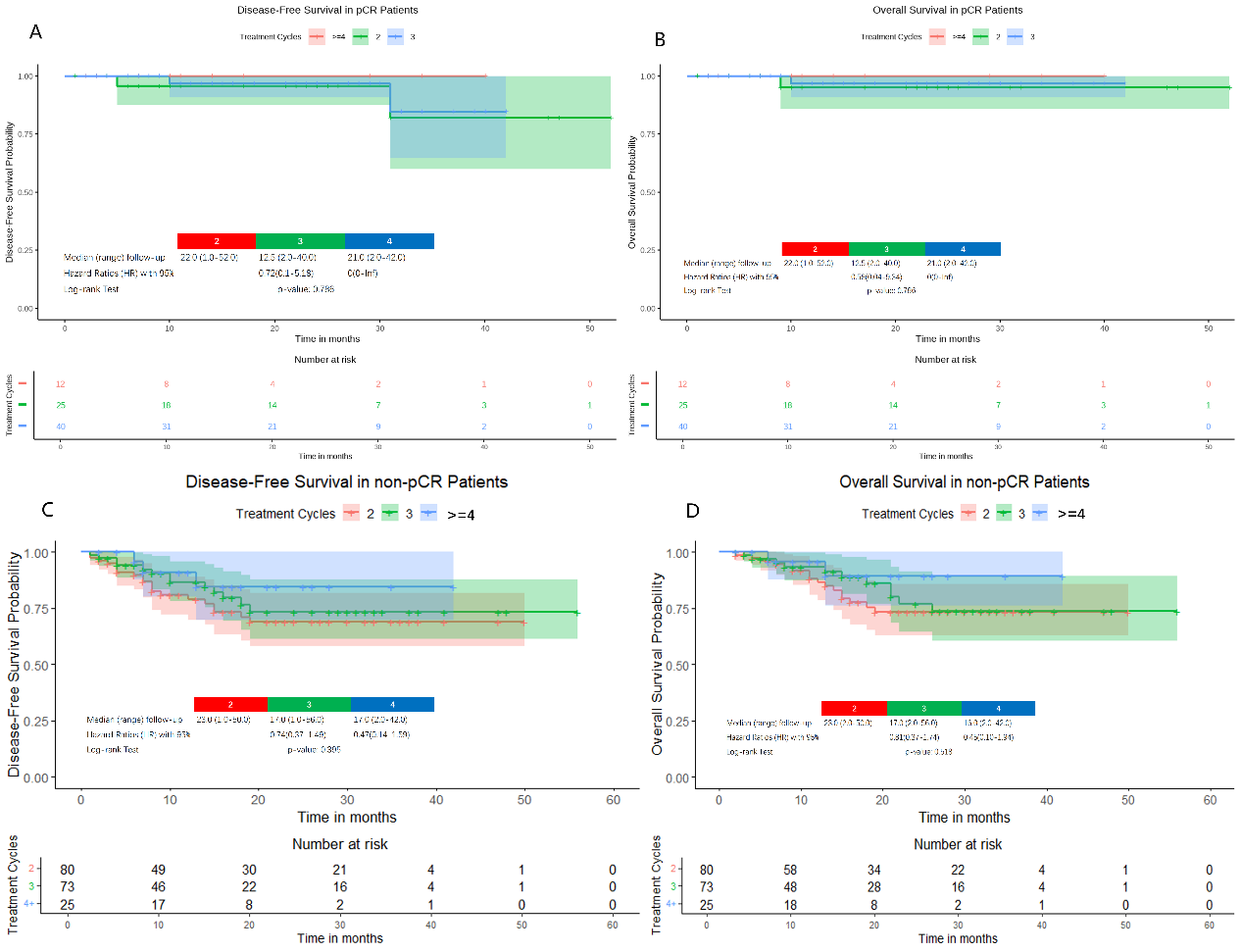

Supplement: Supplementary file 1 [file Table1.docx]
